# From Competence to System-Readiness: Aligning Medical Education With Health System Realities in Low- and Middle-Income Countries

**DOI:** 10.7759/cureus.99112

**Published:** 2025-12-13

**Authors:** Hosam Hefny

**Affiliations:** 1 Medical Education, Faculty of Medicine, Suez University, Suez, EGY

**Keywords:** competency-based medical education, health system-based education, low- and middle-income countries, medical education reform, system-ready physicians

## Abstract

Medical education in low- and middle-income countries (LMICs) often results in clinically competent doctors who are not well equipped to function effectively in fragile, resource-limited health systems. Competency-based medical education (CBME) provides structured learning outcomes, observable competencies, and accountability; however, it was developed mainly in high-income countries with stable health systems and ample resources and thus has limited relevance in the LMIC context. The challenges faced by medical graduates in LMICs include managing scarce resources, leading a multidisciplinary team, conducting quality improvement initiatives, and addressing population-level health priorities, which are capacities we define as system-readiness. Health Care System-Based Medical Education (HCSBME) adds value to CBME by embedding learners in an operational health system setting, such as hospitals, primary care, rural clinics, and community programs, and fosters the development of leadership, teamwork, cost-awareness, and system-level thinking. Integration of CBME and HCSBME enables the medical education system to train physicians who are clinically competent and system-ready. Evidence from Rwanda, Thailand, Ghana, and Ethiopia shows that system-embedded curricula improve workforce distribution, retention, and preparedness for responding to health system needs. This integration thus fills the existing gaps in LMIC medical training and aligns the preparation of physicians with the needs of patients and health systems to improve workforce and health system resilience.

## Editorial

Medical education in low- and middle-income countries (LMICs) has made tremendous progress towards ensuring clinically competent physicians, but evidence shows that graduates are not well prepared to function effectively within complex and resource-constrained health systems [1,2]. While competency-based medical education (CBME) offers a systematic framework that focuses on observable competencies, accountability, and clearly defined learning outcomes [3], it was largely developed and tested in high-income countries characterized by stable health systems and ample resources. Therefore, CBME would not be sufficient to prepare physicians for the realities of the health systems in LMICs, where practitioners need to navigate weak referral networks, manage limited supplies, lead multidisciplinary teams, drive quality improvement initiatives, and consider priorities at the population level. We define system-ready physicians as clinicians who can combine individual clinical competence with the ability to adapt to, strengthen, and innovate within their health systems. Health Care System-Based Medical Education (HCSBME) completes CBME by placing students in the operational contexts of health systems: hospitals, primary care, rural clinics, and community programs-thus fostering leadership, teamwork, cost-awareness, and system-thinking. Integration of CBME with HCSBME enables medical education to align learning objectives not only to patient care but also to the needs of the health system and to produce physicians who can function in the real world while striving to improve the performance of health systems.

This integrated approach is supported by empirical and policy evidence. The Lancet Commission [1] stressed that health-professional education should be aligned with health system needs, especially in LMICs, while WHO guidelines (2013) [2] advocate for placing trainees in service delivery settings to develop system readiness. The evaluation of national programs in Rwanda shows that linking medical curricula to national health priorities leads to improved workforce distribution with a view towards universal health coverage [4]. Thailand’s rural pipeline initiatives demonstrate that placing students in both community and rural settings increases retention and effectiveness in underserved areas [5]. Systematic reviews reveal consistent deficits among LMIC graduates regarding leadership, quality improvement, and interprofessional collaboration, adding weight to the argument that CBME is not alone sufficient to produce physicians ready for health systems [6]. Taken together, these indications suggest that combining CBME with HCSBME is not just conceptually based but also empirically grounded and actually provides a path toward aligning education with the operational imperatives of health systems.

While this integrated model makes intuitive sense, there are some significant challenges to implementing it. These include misalignment of ministries of education and health, limited faculty expertise in systems-based teaching, resource constraints for community placements, and current accreditation standards that emphasize individual clinical competencies at the expense of system-level outcomes. Overcoming these will require coordinated policy action, faculty development, investment in infrastructure, and a redesign of assessment frameworks to evaluate both clinical and system-level competencies. Despite these challenges, there is growing evidence to suggest that LMICs can feasibly adopt integrated curricula through context-specific adaptations and collaborative curriculum design in efforts toward strengthening health system alignment.

By embedding education in health system realities and defining learning outcomes that encompass both clinical and system-level competencies, LMIC medical schools can produce graduates who are skilled not only as clinicians but also as change agents of health system improvement. Such graduates will be leading multidisciplinary teams, optimizing patient flow, managing scarce resources, and implementing quality improvement initiatives to enhance population health outcomes. Integration of CBME with HCSBME will make medical education a strategic investment in health system resilience, ensuring that graduates are prepared for effective responses to system shocks (pandemics or resource crises) while concurrently fostering advances in patient care and population health. The ultimate goal is to create a cadre of physicians as change agents who are outstanding at the bedside and in the health system for sustainable improvements in healthcare delivery and public health outcomes.
